# Targeting CYP450 modulation to decrease the risk of induced cataract in the experimental model

**DOI:** 10.4103/0301-4738.71676

**Published:** 2010

**Authors:** D V Patel, T R Gandhi, K V Patel, D B Patil, P V Parikh

**Affiliations:** Department of Pharmacology, Anand Pharmacy College, SPU, Anand, India; 1 Department of Pharmacology, College of Veterinary Science and Animal Husbandry, AAU, Anand, India

**Keywords:** Cataract, CYP450, galactose, lens

## Abstract

**Background:**

Diabetes is one of the major causes of cataract. Some drugs prescribed for the treatment of diabetes are the modulators of CYP450, which may alter the risk of cataract.

**Objective:**

To study the effect of CYP450 modulation in galactosemic cataract.

**Materials and Methods:**

Male Sprague-Dawley suckling rats were allotted to four groups (n = 6), as follows: Group 1: Normal control, Group 2: Galactose control, Group 3: CYP450 inhibitor pretreated and Group 4: CYP450 inducer pretreated. Cataract was induced in animals of all groups except group 1 by feeding them galactose (50%), 21 days after parturition. From the eighteenth day of life, CYP450 inhibitor (nifedipine; 8.1 mg/kg) and CYP450 inducer (pioglitazone; 3.8 mg/kg) were given orally to groups 3 and 4, respectively. The maturation pattern of the cataract was observed by an operating microscope, every third day. Biochemical changes in the lenses of all groups, for example, CYP450 activity expressed as µM NADPH oxidized / unit time, alterations in the levels of total proteins, soluble proteins, and reduced glutathione (GSH) following the induction of cataract, were estimated.

**Results:**

The microscopic examination of the lenses indicated that CYP450 inhibitor pre-treatment delayed (fourteenth day) the occurrence of cataract, while CYP450 inducer pretreatment demonstrated an early (ninth day) cataract as compared to galactose control rats (twelfth day). A significant decrease and increase in CYP450 activity was observed with the CYP450 inhibitor and inducer pre-treatment, respectively. There was no alteration in the GSH level, but a significant increase in total and soluble protein was found in groups 3 and 4 as compared to group 2.

**Conclusion:**

CYP450 may have a role in the initiation of cataract without any effect on the maturation pattern, as revealed by the delayed occurrence of cataract with the CYP450 inhibitor and an early onset of cataract with the CYP450 inducer.

A cataract is a clouding of the natural lens, the part of the eye responsible for focusing light and producing clear, sharp images. The cataract remains the leading cause of visual disability and blindness all over the globe.[1,2] Systemic diseases (such as diabetes, gout, and arthritis) are associated with cataract.[3,4] The proportion of cataract patients with diabetes has been found to range from 8.7 to 21%, which is considerably greater than the prevalence in the general population, which shows a high risk of cataract associated with diabetes.[5] Evidence has accumulated for the involvement of polyol metabolism and the enzyme aldose reductase in diabetic cataractogenesis.[6,7] Cataract formation in diabetics is attributed to the accumulation of sorbitol in the lens. The formation of sorbitol in the lens is due to the conversion of excess glucose to sorbitol by the enzyme aldose reductase using nicotinamide adenine dinucleotide phosphate (NADPH) as a cofactor.[7,8] The electron transfer from NADPH further depends on the cytochrome P450 (CYP450) system. A typical cytochrome P450 catalyzed reaction is:

$$\mathrm{NADPH}\;+\;H^{+}\;+\;O_{2}\;+\;\mathrm{RH}\;{\mathrm{NADP}}^{+}\;+\;H_{2}O\;+\;R-\mathrm{OH}\;$$

CYP450 reductase transfers electrons from NADPH to the various isoforms of CYP450. This means by inducing or inhibiting CYP450 one can alter the activity of the aldose reductase and thus sorbitol is formed.

It is a well-known fact that aging is one of the major risk factors for various diseases such as hypertension and diabetes. Such patients are often treated with antidiabetic and antihypertensive agents, some of which are CYP450 modulators. Thus, this study has been undertaken to evaluate the effect of a CYP450 inducer (pioglitazone) and a CYP450 inhibitor (nifedipine) on the occurrence of cataract.

## Materials and Methods

Male Sprague-Dawley rats (age 18 days) weighing 40 – 50 g were randomly divided into four groups (n = 6 for each group): Group 1 (normal control), Group 2 (galactosemic rats treated with vehicle solution), Group 3 (galactosemic rats treated with CYP450 inhibitor-nifedipine), Group 4 (galactosemic rats treated with CYP450 inducer-pioglitazone). The animals were housed at an ambient temperature (25 ± 10°C) and relative humidity (55 ± 5%) and 12 hours / 12 hours light / dark cycle. Animals had free access to a standard pellet diet and water was given *ad libitum*. The experimental protocol was approved by the institutional animal ethical committee (IAEC), as per the guidelines of the committee for the purpose of control and supervision of experiments on animals (CPCSEA), Ministry of social justice and empowerment, Government of India (Protocol No. project 6005 dated 20^th^ November, 2006).

Cataract formation was induced by feeding galactose to suckling rats.[9] The composition of the diet was galactose (50%), cornstarch (20%), casein (15%), hydrogenated oil (9%), salt mixture (1.4%), and cod liver oil (2%). All animals were checked daily during the experiments for the appearance of cataract. The eyes were examined with an operating microscope (OM-18; magnification 6X) to observe the maturity of cataractogenesis, every third day from galactose feeding. Clinically, the nuclear opacities were graded from one to four according to the severity of the opacity.[10] The experiment was continued until all the lenses were affected with cataract. The eyes were enucleated under ether anesthesia or the lenses were dissected free and kept in a cell culture dish with chilled phosphate buffer (pH-7) (prepared as per IP’96).

Pretreatment with nifedipine (8.1 mg/kg; P.O; once daily) and pioglitazone (3.8 mg/kg; P.O; once daily) was started on day 18 after parturition for the animals of groups 3 and 4, respectively. The animals of groups 2, 3, and 4 were fed with a galactose-rich diet starting from day 21 after parturition and the animals of group 1 were fed *ad libitum*.

The maturation pattern was examined and the following biochemical parameters were estimated: protein contents,[11] CYP450 activity,[12] and reduced glutathione.[13] The results were expressed as mean ± SEM. Statistical analysis was carried out using the ANOVA followed by Post Hoc TUCKEY. A *p* value of ≤0.05 was considered to be statistically significant.

## Result

The gross examination indicated that the animals of group 2 had the appearance of cataract on the thirteenth day of galactose feeding, whereas, cataract was seen on day 14 in group 3, and day 11 in group 4. From day 15 of galactose feeding, the difference in appearance of cataract was significant between all the groups (*P* < 0.05). Pretreatment with nifedipine delayed the onset of galactose- induced cataract, as the cataract was not visualized in the lenses of this group on the thirteenth day of galactose feeding, as against three lenses in the galactose control group, which had the appearance of cataract on the same day. Contradictory to that, pioglitazone pretreatment demonstrated an early appearance of cataract as evidenced by one cataractous lens on day 11 of galactose feeding, as against no cataractous lenses in the galactose control animals [Table 1].

**Table 1:** Progression of cataract (gross examination)

nth day of galactose feeding	No. of lenses affected by cataract		
	Group 2	Group 3	Group 4
11	0.0	0.0	1.0
12	0.0	0.0	2.0
13	3.0	0.0	5.0
14	7.0	2.0	6.0
15	12.0*	5.0*	6.0*
16	12.0*	7.0*	7.0*
17	12.0*	9.0*	10.0*
18	12.0*	12.0*	12.0*

* ANOVA followed by post hoc TUCKEY concluded a significant difference (*P* ≤ 0.05) in progression of cataract within all groups

**Figure 1:**
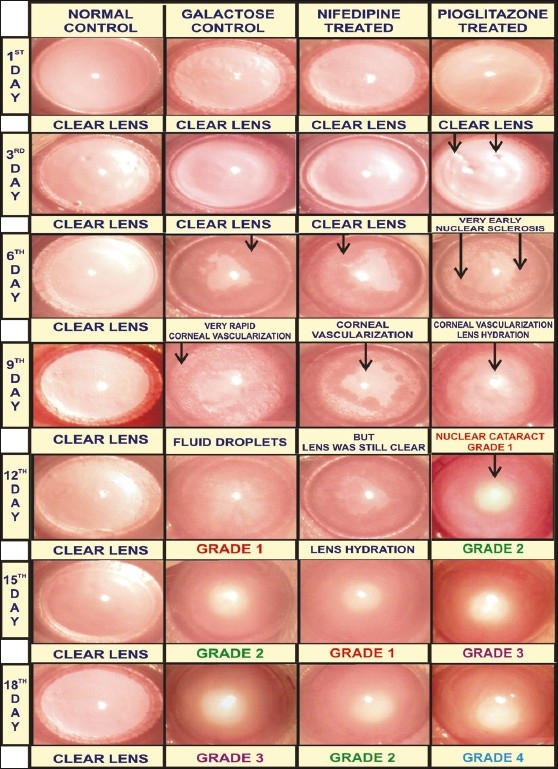
Microscopic Evaluation of Cataract. Photographs of lenses using Operating Microscope (OM-18; magnification 6X). (Group 1) Normal control; (Group 2) Galactose control; (Group 3) CYP450 inhibitor-nifedipine pretreated galactosemic lens; (Group 4) CYP450 inducer-pioglitazone pretreated galactosemic lens

The above-mentioned observations were supported by the microscopic examination of the lens, which revealed the significant difference in occurrence of cataract in various groups. Rapid corneal vascularization followed by fluid droplets (hydration of lenses) was observed in the initial period of galactose feeding (within six to nine days, Fig. 1). Similar changes were observed early in group 4 (within three to six days, Fig. 1) and delayed in group 3 (within nine to 12 days, Fig. 1). Corneal vascularization was observed in galactose-induced cataract (sixth day, Fig. 1) and this change disappeared soon after the formation of the nuclear cataract. The nifedipine pretreatment in group 3 delayed the occurrence of cataract as revealed by nuclear cataract grade 1 on the fourteenth day of galactose feeding as compared to that on day 12 in group 2. Pioglitazone pretreatment in group 4 resulted in the early occurrence of cataract as revealed by nuclear cataract grade 1 on the ninth day of galactose feeding as compared to that on the twelfth day, in group 2 [Fig. 1].

**Figure 2:**
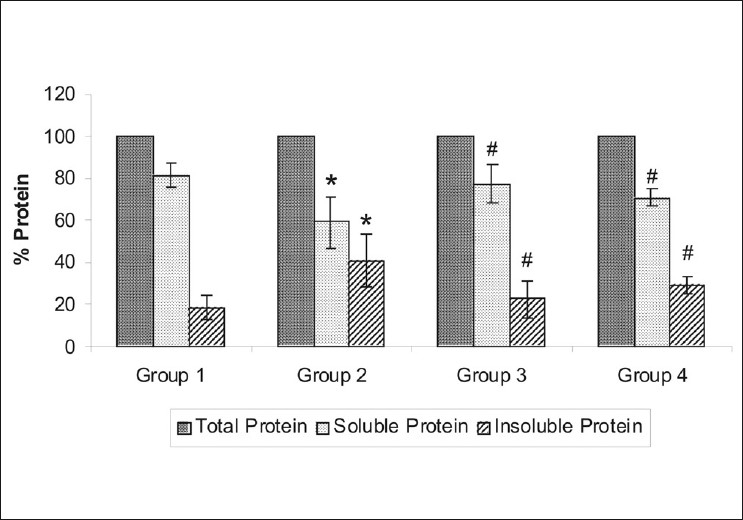
Plots of relative protein contents from (Group 1) Normal control; (Group 2) Galactose control; (Group 3) CYP450 inhibitornifedipine pretreated galactosemic lens; and (Group 4) CYP450 inducer-pioglitazone pretreated galactosemic lens. The vertical lines inside the plot represent the SEM. *P* ≤ 0.05: *versus group 1, #versus group 2

The total proteins, that is, the soluble proteins and insoluble proteins were estimated [Table 2], and the relative protein content was also calculated. In group 2, a significant decrease in soluble proteins (58.94 ± 12.84%) and an increase in insoluble proteins (41.06 ± 12.84%) was observed as compared to group 1, wherein, there were 81.40 ± 5.91% soluble proteins and 18.60 ± 5.91% insoluble proteins (*P* = 0.0035) [Fig. 2]. In case of group 3, the soluble proteins and insoluble proteins were 77.40 ± 9.20% and 22.60 ± 9.20%, respectively, and in group 4 the soluble proteins were 70.84 ± 3.96% and insoluble proteins were 29.16 ± 3.96% [Fig. 2]. The magnitude of decrease in the total protein level (572.26 ± 84.77µg/ml) was noted to be significant in the lenses of group 2, as compared to the lenses of group 1 (1999.97 ± 329.7 µg/ml) (*P* = 0.002). However, total proteins in the lenses of group 3 (1587.69 ± 142.96 µg/ml) and group 4 (928.71 ± 81.48 µg/ml) were significantly higher than those in group 2 (572.26 ± 84.77 µg/ml) (*P* < 0.001 and *P*= 0.0018).

**Table 2:** Biochemical values of studied parameter

Parameters	Group 1	Group 2	Group 3	Group 4
Total protein (µg/ml)	1999.97 ± 329.7	572.26 ± 84.77*	1587.69 ± 142.96##	928.71 ± 81.48#
Soluble protein (µg/ml)	1692.73 ± 361.39	303.17 ± 60.12*	1209.23 ± 134.74##	652.13 ± 58.09#
Insoluble protein (µg/ml)	307.24 ± 83.75	269.07 ± 67.32	378.46 ± 154.39	276.20 ± 55.22

From (Group 1) Normal control; (Group 2) Galactose control; (Group 3) CYP450 inhibitor-nifedipine pretreated galactosemic lens; (Group 4) CYP450 inducerpioglitazone pretreated galactosemic lens. *P* ≤ 0.05:

* versus group 1

** versus group 1

# versus group 2, *P* ≤ 0.001:

## versus group 2

**Figure 3:**
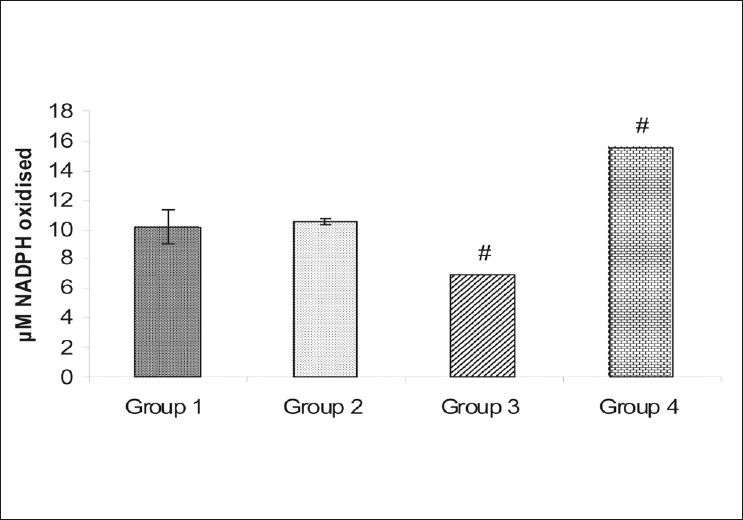
Plots of CYP450 activity expressed as µM NADPH +/5 minutes from (Group 1) Normal control; (Group 2) Galactose control; (Group 3) CYP450 inhibitor-nifedipine pretreated galactosemic lens;(Group 4) CYP450 inducer-pioglitazone pretreated galactosemic lens. The vertical lines inside the plots represent the SEM. *P* ≤ 0.05: *versus group 1, #versus group 2

The activity of CYP450, expressed as µM NADPH oxidized/unit time, after 5 minutes was 10.17 ± 1.20 µM in case of group 1 and 10.51 ± 0.17 µM in case of group 2. A significant inhibition and induction of CYP450 activity was observed in group 3 (6.89 ± 0.0µM) (*P* = 0.002) and group 4 (15.51 ± 0.0 µM) (*P*=0.0011), respectively [Fig. 3].

**Figure 4:**
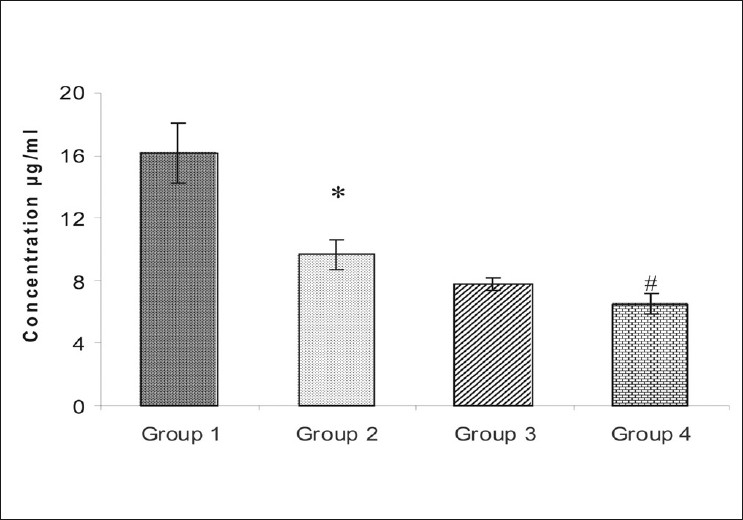
Plot of reduced glutathione content of the lens from (Group 1) Normal control; (Group 2) Galactose control; (Group 3) CYP450 inhibitor-nifedipine pretreated galactosemic lens; and (Group 4) CYP450 inducer-pioglitazone pretreated galactosemic lens. The vertical lines inside the plots represent the SEM. *P* ≤ 0.05: *versus group 1, #versus group 2

A significant decline in lens glutathione (GSH) content was observed in group 2 as compared to group 1. However, no significant change in GSH content was observed in groups 3 and 4 [Fig. 4], as compared to group 2.

## Discussion

The prevalence of cataract in persons with diabetes has been found to be greater than its prevalence in the general population, which shows the high threat of cataract associated with diabetes.[5] The present study incorporated a galactose-rich (50%) diet to induce cataract. It is believed that the key event in galactose-induced diabetic cataractogenesis is the activation of the polyol pathway, with conversion of galactose into the subsequent polyols, such as, sorbitol, dulcitol, and galactitol, by the aldose reductase enzyme. This conversion (in sugar cataract) by aldose reductase requires NADPH as a cofactor, which in turn requires cytochrome for an electron transfer.[14] The resultant polyols are unable to escape from the lens and are readily metabolized, and therefore, they accumulate within the lens. This creates severe osmotic stress, leading to swelling and loss of integrity.[15] On this basis, a hypothesis can be put forth, stating that by inhibiting or inducing CYP450 one may alter the polyol pathway and hence the occurrence of cataract.

In the present study, the gross examination of the lenses of animals fed with a galactose diet in group 2 showed the appearance of cataract after 13 days of feeding. The appearance of cataract in the lenses was observed on the fourteenth day in group 3 and on the eleventh day in group 4. This demonstrates that nifedipine delays and pioglitazone accelerates the appearance of cataract as compared to the control group. However, at the end of an 18-day study, cataract was observed in all the animals of both test groups on gross examination of the lenses. Thus, it can be concluded that the CYP450 inhibitor can delay and the CYP450 inducer can accelerate the appearance of cataract in the galactose-induced model.

Microscopic evaluation of the lenses indicated a similar maturation pattern of the cataract in all groups. The CYP450 modulator did not bring about any alteration in the maturation pattern of the cataract. However, the time required for maturation was altered. This is evident from the occurrence of a nuclear cataract grade 1 on the ninth and fourteenth day in groups 3 and 4, respectively [Fig. 1]. The microscopic examination implies that the CYP450 modulators have a significant effect on the occurrence of cataract without significantly altering the maturation pattern.

As compared to group 2, the level of NADPH after five minutes was significantly higher in group 3 and significantly lower in group 4, which indicates the inhibition and induction of the CYP450 activity (calculated as µM NADPH oxidized/5 minutes) in groups 3 and 4, respectively. No difference was observed in the activity of CYP450 between groups 1 and 2, suggesting no effect on the CYP450 activity by galactose feeding.

Galactose-induced cataract results in the formation of polyol, that is, sorbitol, which is neither able to escape from the lens nor is it readily metabolized. This induces a large movement of water into the lens, causing alteration of the Na^+^/K^+^ ratio and concomitant lens swelling. The normal lenticular architecture is thus disrupted, and the soluble proteins are decreased. According to this hypothesis, the non-enzymatic glycosylation (glycation) of the lens proteins produces adducts that play an important role in the causation of sugar-induced cataracts.[6,16] The initial reaction is the attack of an open-chain form of the sugar on the amino groups of the lens proteins. The initial attack leads to the formation of a variety of chemical entities and induces structural changes in the enzymes, membrane proteins, and crystallins in the lens. Thus, soluble proteins are decreased and insoluble proteins are increased. This leads to hyperosmotic cell swelling, which produces a scattering of light and diminished lens transparency.[6,17,18] GALACTOSEMIC cataract formation is also associated with altered lens GSH homeostasis.[19,20]

In this study, significant changes in the lens proteins and GSH level of galactose control group were observed as compared to the normal control rats. Feeding of galactose significantly decreased the soluble proteins and GSH level and increased the insoluble proteins of the lens as compared to the normal control animals, which resulted in cataract. Pretreatment with nifedipine significantly prevented galactose-induced reduction in the total proteins and soluble proteins. As pioglitazone was a CYP450 inducer, it should have the opposite action on proteins. This action can be attributed to the alteration in the galactose-induced polyol pathway through CYP450 modulation or galactose-induced alteration in GSH homeostasis. Pretreatment with nifedipine and pioglitazone did not show any antioxidant effect, as no significant change in the GSH content in the lenses of group 3 and 4 was seen, as compared to group 2.

Thus, the results reveal that inhibition of CYP450 activity may affect the polyol pathway and delay the appearance of cataract, increase the soluble proteins, lessen the area opacity, and vice versa. Also nifedipine (CYP450 inhibitor) has no significant effect on the glutathione content. Cominacini *et al*. have reported that nifedipine has no effect on ROS formation.[21] Thus, nifedipine delays the appearance of galactose-induced cataract, by inhibiting the CYP450 enzyme only. p0 ioglitazone (CYP450 inducer) has not shown any effect on the glutathione content, so the early appearance of cataract may be due to its inducing effect on the CYP450 enzyme.

Further investigations using a variety of CYP450 modulators and estimation of antioxidant activity, are needed to identify the pharmacological profile of CYP450 modulators on cataractogenesis.

## Conclusion

CYP450 may have role in the initiation of cataract, without any effect on the maturation pattern, as revealed by the delayed occurrence of cataract with the CYP450 inhibitor and an early onset of cataract with the CYP450 inducer in the experimental rat model.
